# Atlanta Society of Medicine

**Published:** 1897-09

**Authors:** Thos. H. Hancock

**Affiliations:** Secretary; Atlanta


### Society Reports.

### ATLANTA SOCIETY OF MEDICINE.

ATLANTA, July 20, 1897.

The meeting was called to order by the President, Dr. G. H. Noble.

The following members were present: Drs. M. G. Campbell, Murphy, Todd, Kendrick, Smith, Duncan, Hood, J. McF. Gaston, Jr., and Hancock.

The minutes of the previous meeting were read and approved.

DR. W. S. KENDRICK made the following remarks on scarlet fever: All of the eruptive diseases are ushered in by various conditions, etc. The specific cause differs in the different diseases. In scarlet fever the eruption is due to a specific cause. We have also a specific cause in measles, whooping-cough, pneumonia, typhoid fever, etc. The exact cause is not known. It is supposed to be due to a streptococcus by some, but it is not definitely determined. Scarlet fever is an eruptive disease. The agent producing it is present from the beginning to the end. It is largely conveyed in the scales which come off from the skin. The feeling is due to a dermatitis.

In slight attacks it is not so abundant. These are some of the conditions. It usually occurs in those under ten years of age and over twelve months old. In very young subjects it is very fatal. It may attack adults, but this is not the rule. Probably the reason grown people do not have it is, that one attack makes them immune and most of them have had it in childhood. It is more prevalent in certain seasons of the year. There are always some sporadic cases in large cities. About every five or seven years we have a larger percentage than in the interval.

### PATHOLOGY.

We have a true dermatitis, inflammation of the lymphatics, dilatation of the blood vessels with exudation of serum and afterwards shedding of the epidermis.

*Symptoms:* The onset is usually sudden (not so in measles); the child may go to bed at night well, and wake up with nausea and vomiting or even convulsions. Those cases which commence with convulsions are always very severe. This onset is unlike that of measles, which has a prodroma with fever for a day or two and at the time of the eruption a second elevation of temperature. Scarlet fever, I have said, comes on suddenly.

In about thirty-six hours usually we have the eruption, beginning on the neck, then extending down on the shoulders, and upon the face. Usually in twenty-four to forty-eight hours the pulse-beat is from one hundred and twenty to a hundred and fifty. The fever is usually at about its height in two days. This lasts about thirty-six hours, and then declines and the symptoms disappear. We usually have a high fever, rapid pulse, and inflammation of the throat. It is very variable, however. In some epidemics almost all the cases get well, and in others a great many die. In mild cases there is often only a congestion of the throat, but in severe cases, where the temperature is a hundred and three or four, the rash is more pronounced. The throat symptoms are more aggravated and we have follicular tonsilitis with inflammation about the fauces, palate, etc. There is another variety of cases where we have graver throat symptoms and worse constitutional trouble. In these we have a pseudomembranous angina. It is hard to tell whether it is true scarlet fever or diphtheria or a combination of the two. We may have a rash with diphtheria. The rash in these severe cases of scarlet fever is confined to the trunk of the body, as a rule. I once had a case in a young lady patient. Her pulse was a hundred and fifty and her temperature a hundred and five. I saw her two or three times a day and this same condition lasted four or five days. The pulse was rather feeble. Dr. Harris examined the deposits of the throat and he found evidences of diphtheria, bacilli, etc It was not a typical case. He thought it was a mixed infection. She had desquamation, but it was not marked. She also had a new deposit on one tonsil and had a very hard time, being confined to her bed five or six weeks. We may have an eruption in true diphtheria and we may have a false membrane in scarlet fever. I have not had a great many cases of scarlet fever. I think that sometimes other things are taken for it. We may have a rash in follicular tonsilitis and may even have desquamation. We may have a rash from many causes, as entero-colitis, indigestion, etc. In scarlet fever the throat symptoms are usually bilateral. In tonsilitis it is only on one side as a rule. That is, in severe cases where an abscess forms. One tonsil in the case of the young lady I mentioned was only one-third the size of the other. I did not think it was scarlet fever at first. She had desquamation, however, and other symptoms afterwards which convinced me that it was. We have a rash also in septicemia, which I failed to mention above. The epidemics are very variable. Some are grave and some mild. In some we lose no case in twenty or thirty cases, and in others we lose one-third of all the cases we have We may have hemorrhages in various places, and these are always grave cases. Some are malignant, and succumb in from thirty-six to forty-eight hours. Some of the cases present grave nervous types. The desquamation usually begins from the seventh to the tenth day. In a few cases it may begin before the eruption disappears. It may end in ten or fifteen days, and it may last six weeks. There may be a second desquamation. In some cases the nails, soles of the feet, and palms of the hand peel off. The skin of the hands may come off like a glove. The greatest contagion is during desquamation. It may be catching before the eruption appears, but is not so liable. The germs may remain potent for weeks or months. Hence disinfection should be thorough.

*Diagnosis:* The diagnosis is easy as a rule. In the same family we may have two or three cases which are typical and others that are not at all so. We may have scarlet fever *sine eruptione,* in which we have the constitutional symptoms and not the others. All the authorities agree on this point. The differential diagnosis has already been mentioned. It takes four or five days to tell whether the case is scarlet fever or measles. The other diseases which may be confounded with it, I will not mention. When the inflammation goes up in the posterior nares or down in the larynx it is always grave, and nineteen cases out of twenty die, whether inflammation is due to scarlet fever or other complications.

*Otitis, Suppurative:* The inflammation may extend up the eustachian tube, into the middle ear. This is the main cause of deafness in children.

*Cardiac Complication:* Valvular disease of the heart, as mitral or aortic lesions, may be due to scarlet fever. Many of these cases are due to it and remain latent until later in life. I have seen many of them in my clinics and in my private practice. *Nephritis* is the most common and dangerous of all the complications it usually occurs two or three weeks after the eruption ceases. There may be dropsy. In severe cases there is little turbid bloody urine which contains casts, albumen, etc. Even in mild attacks we may have the severest forms of nephritis. Some of the cases die in twenty-four to thirty-six hours after it develops.

### TREATMENT.

It is a self-limited disease. Mild cases require little treatment. Keep the bowels active, bathe the patients frequently in warm water and keep them well oiled to allay the itching and prevent the scales from flying about. Treat the otitis and nephritis as you would in other cases. In the grave cases of scarlet fever use cold water instead of the coal tar products for the fever. There is nothing better than cold water or tepid water baths for fever. Keep all of them isolated and in bed as long as possible. They should be kept in their room from three to six week. Keep them in bed to avoid nephritis. I beg your pardon for talking so long. I have not covered the field, but have just given a general outline.

DR. TODD: The text-books do not give us a good clinical history of this disease. In some epidemics, as Dr. Kendrick has said, they nearly all get well, and in others a great many die. I have not seen as many cases as some of the other doctors here. I think some of them make wrong diagnosis. In la grippe there may be an eruption. Quinine also causes an eruption in some cases. This is not very frequent, however. The similarity between scarlet fever and diphtheria is so great sometimes I hoped that Dr. Kendrick would give us a positive differential diagnosis. I think that many have both contagions at the same time. The books, especially those printed in Europe and in the North, say there is nothing so contagious as scarlet fever. This is not so in my experience. Some cases are very contagious and others are not at all so. In my own family there were two very slight cases, but in the beginning we can not tell what sort of complications will occur. Both of my children had nephritis and my boy had almost complete paralysis in his legs. My sister died about this time and left her children to me. Dr. Harris was then about thirteen years old. They were all brought to my house, and Dr. Harris was the only one who had scarlet fever. My niece visited my house and my wife told her not to come in, but she did and carried it Dr. Ridley's and also to her own home. The word scarlatina does much harm, as we may get the worst form of scarlet fever from it. There are some curious freaks. I remember a family where the oldest child had a bad case and there were four other children in the house. There was no isolation. Among the five from the oldest to the youngest child it was eleven months before they all had it. There are more malignant cases in the North than there are here. In the Perkins family, here in Atlanta, there were three cases, the mother and two children, who died in thirty-six hours after the onset. They were overwhelmed with the poison. A curious thing in these cases was, that their pulses were not affected by it. I believe, in the malignant cases with the bad throat, that serum therapy may do some good. The strawberry tongue does not occur until the third or fourth day, as a rule. The books lead you to believe that this is one of the first symptoms. Adenitis may be aborted by applying ice in a bag to the affected area and I have never seen one suppurate where it was used. I do not know whether it is the greater dose or the greater resistance of the individual, which makes the bad cases. I saw two cases in one family where one child died in thirty-six hours after the onset and the other child had a very mild case, requiring no treatment, and had no sequelæ. There must be a difference in the resistance of different individuals. There is no doubt that some epidemics are worse than others. If the temperature is over a hundred and two I put them in a warm bath and cool it down. In the last five or six years I

have had no death from scarlet fever. I think the cold water kept off the sequelæ. Tub baths are the best, but you may use the wet pack or sponge baths. Nephritis may be fatal, or may require long treatment. Where we have parenchymatous nephritis fifty per cent. of the cases are fatal. Any diuretic is not indicated. We should not work a sick horse. Then the skin, etc., should do the work. I agree with Dr. Kendrick about not giving antipyretics.

DR. DUNCAN: I thank Dr. Kendrick and Dr. Todd both for their able handling of this subject. I agree about the mildness of some of the epidemics, and the malignancy of some others. Also about not giving the antipyretics. I use jaborandi or pilocarpin in the acute stage or in case of nephritis to eliminate the poison by the skin. I believe in the baths, also. I had a case in a boy three years old. A younger brother had had a mild attack, and I did not want to put up the scarlet fever card, but I did and in three nights the three-year-old boy had it and died in thirty-six hours. We may get a malignant case from a mild one, or a mild one from a malignant one. I am skeptical about the contagion of scarlet fever. In some families they all have it, and in others only one member. I believe the children get the disease from impure water. I never had a case that did not drink well water. I use oil of eucalyptus and oil of vaseline for the skin. The oil of eucalyptus is a disinfectant. I also use the baths or wet pack or sponge baths. I do not agree with Dr. Todd about diuretics.

DR. GASTON, JR.: I have only a few words to say about scarlet fever. In the special cases mentioned there is no word that expresses as well as poison. In some cases the system seems to be overwhelmed with it. I like pilocarpin and jaborandi. They make the sweat glands do the work of the kidney. I like to use them by hypodermic injection, as some of them are not able to retain drugs taken internally. If they can retain the drugs internally, they generally do well. I endorse the water treatment and the diaphoretics. If I am in doubt about the diagnosis I treat the case as if it were scarlet fever anyway.

DR. MURPHY: I have noticed in regard to the vomiting, if it comes at the beginning, it is usually a fatal case. I saw a case that had nephritis. They said it had not had scarlet fever, but it had evidently had it very slightly, and it died in a year.

DR. M. G. CAMPBELL: I have recently treated six cases, four of which were in one house. I was called at night to see the family, which were negroes, and I did not recognize the disease that night. The next evening one of the children was dead. In one case the epidermis peeled on the abdomen, etc. There were white streaks in the skin, and the pigment has not yet returned. I find that carbolic acid salve acts well in the glandular troubles. I use hypodermic injections of Edson's aseptolin, which contains pilocarpin, carbolic acid, etc. This acts well in tubercular troubles also. I also use phenacetin, whiskey and baths. The phenacetin acts nicely on the skin. I use digitalis, acetate of potassium and hot baths for nephritis. I use both diaphoretics and diuretics in these cases.

DR. TODD (second discussion by permission of the chair): Mrs. M. P. Jacobi has shown that the too free use of chlorate of potash caused nephritis. Rheumatism and tonsilitis go together. You must watch the heart as well as the kidneys in these cases. Campho-phenique is good for the itching.

DR. KENDRICK (closing): I have taken much interest in the discussion of this subject. I left out a great many points. I agree with Dr. Todd and Dr. Duncan in regard to the contagion of this disease. I use every precaution to isolate them. Some of the cases are not very contagious. I had a baby downstairs at my house, and a scarlet fever case upstairs and the baby did not contract it. In reference to Dr. Duncan about drinking water, the trend of opinion of all the authorities, is that the disease was given by one person to another, through the scale. These may get into milk or water. Probably the contagion is conveyed by desquamation. In regard to diphtheria and scarlet fever I can usually make a differential diagnosis. In some cases I can not say which it is or whether they are not both present at the same time. The invasion is not sudden in diphtheria. Then the appearance of false membrane, etc., usually enables me to diagnosis the case. In reference to Dr. Murphy about vomiting early in the disease: Whenever the case is ushered in by vomiting or convulsions it is always a grave case.

THOS. H. HANCOCK, Secretary.

